# Microcirculation dysfunction and cardioprotection in cardiac surgery with cardiopulmonary bypass: mechanisms, monitoring, and therapeutic strategies

**DOI:** 10.3389/fcvm.2026.1843167

**Published:** 2026-06-05

**Authors:** Clark Zheng, Kelsey Muir, Himanshu Kaushik, Keertana Yalamanchili, Keyana Zahiri, Adam Yeo, Justin Kim, Frank W. Sellke

**Affiliations:** 1 Department of Surgery, Division of Cardiothoracic Surgery, Warren Alpert Medical School of Brown University, Providence, RI, United States; 2 Cardiovascular Research Center, Department of Surgery, Rhode Island Hospital, Providence, RI, United States

**Keywords:** cardiopulmonary bypass, hemodilution, microcirculation, microcirculatory dysfunction, microvessel

## Abstract

Cardiopulmonary bypass (CPB) is a crucial component of cardiac surgery, yet postoperative organ dysfunction remains common despite apparently adequate systemic hemodynamics. This mismatch underscores the importance of the microcirculation, where oxygen delivery and extraction ultimately occur. Increasing evidence suggests that CPB disrupts capillary perfusion through endothelial dysfunction, glycocalyx shedding, inflammation, oxidative stress, hemodilution, functional shunting, and microthromboemboli, thereby causing tissue hypoxia despite normal macro-hemodynamic stability. This review summarizes the physiology of microcirculation and examines the mechanisms by which CPB impairs microvascular function. We also review current tools for microcirculatory assessment, including orthogonal polarization spectral, sidestream dark field, and incident dark field imaging, near-infrared spectroscopy, and circulating biomarkers of endothelial and glycocalyx injury. Although these approaches have improved mechanistic insight, their clinical use remains limited by technical challenges and the lack of standardized endpoints. We further evaluate cardioprotective strategies aimed at preserving microcirculatory function during CPB. These include perfusion and mechanical approaches, ischemic preconditioning, glycocalyx-preserving therapies, and various pharmacologic interventions such as volatile anesthetics, nitric oxide, statins, antioxidants, and antithrombotic agents. Across these domains, preclinical studies often support biologic plausibility, whereas clinical evidence remains heterogeneous and inconclusive and frequently relies on indirect evidence of organ dysfunction rather than direct measures of microvascular perfusion. Important gaps remain in translating findings from experimental models to patients, and greater integration of bedside imaging and biomarkers with biophysiological principles may help establish microcirculation as both a mechanistic endpoint and a therapeutic target in cardiac surgery.

## 1 Introduction

Over 900,000 cardiac surgery operations are performed in the US each year. The majority of these cases are done with the heart on cardiopulmonary bypass (CPB) with cardioplegia, allowing for a motionless, bloodless operating field (1). Despite major advances in surgical techniques and cardiopulmonary bypass strategies aimed at optimizing macro-hemodynamics such as mean arterial pressure and cardiac output, morbidity after operations with CPB remains substantial. Prolonged ICU and hospital lengths of stay, sepsis, and development of subsequent long-term arrhythmias, cardiopulmonary, neurologic, and renal complications are not uncommon postoperatively, particularly in cases with prolonged CPB times (2).

Although anesthesia and perfusion teams have implemented cardioprotective strategies to optimize macro-hemodynamics, increasing evidence suggests that microcirculation, which is often beyond control with bypass, is thought to be a significant driver of postoperative morbidity. Prior work has demonstrated substantial vascular dysfunction associated with CPB and cardioplegia, often at the level of the microcirculation (3). This in part is attributed to the activation of the coagulation cascade, complement pathways, and other inflammatory mediators and interleukin due to exposure of blood to shear forces from roller pumps and tubing in bypass machine circuitry (4–6). Subsequently, these factors along with hemodilution, hypothermia, and ischemia-reperfusion injury during cardiac surgery and bypass, lead to a mismatch between systemic and microvascular perfusion, leading to heterogeneous capillary perfusion, shunting, poor oxygenation at the microlevel, and ultimately tissue injury.

With an increasingly aging US population, the demand for and volume of cardiac surgeries remain high. The increasing burden of prolonged hospital and ICU stays and management of longer-term postoperative complications has both clinical and economic implications. Directing focus towards further optimizing cardiopulmonary bypass strategies by targeting the microcirculation may offer a new pathway to improve postoperative cardiac surgery outcomes.

## 2 Physiology of cardiac microcirculation

**Figure 1:**
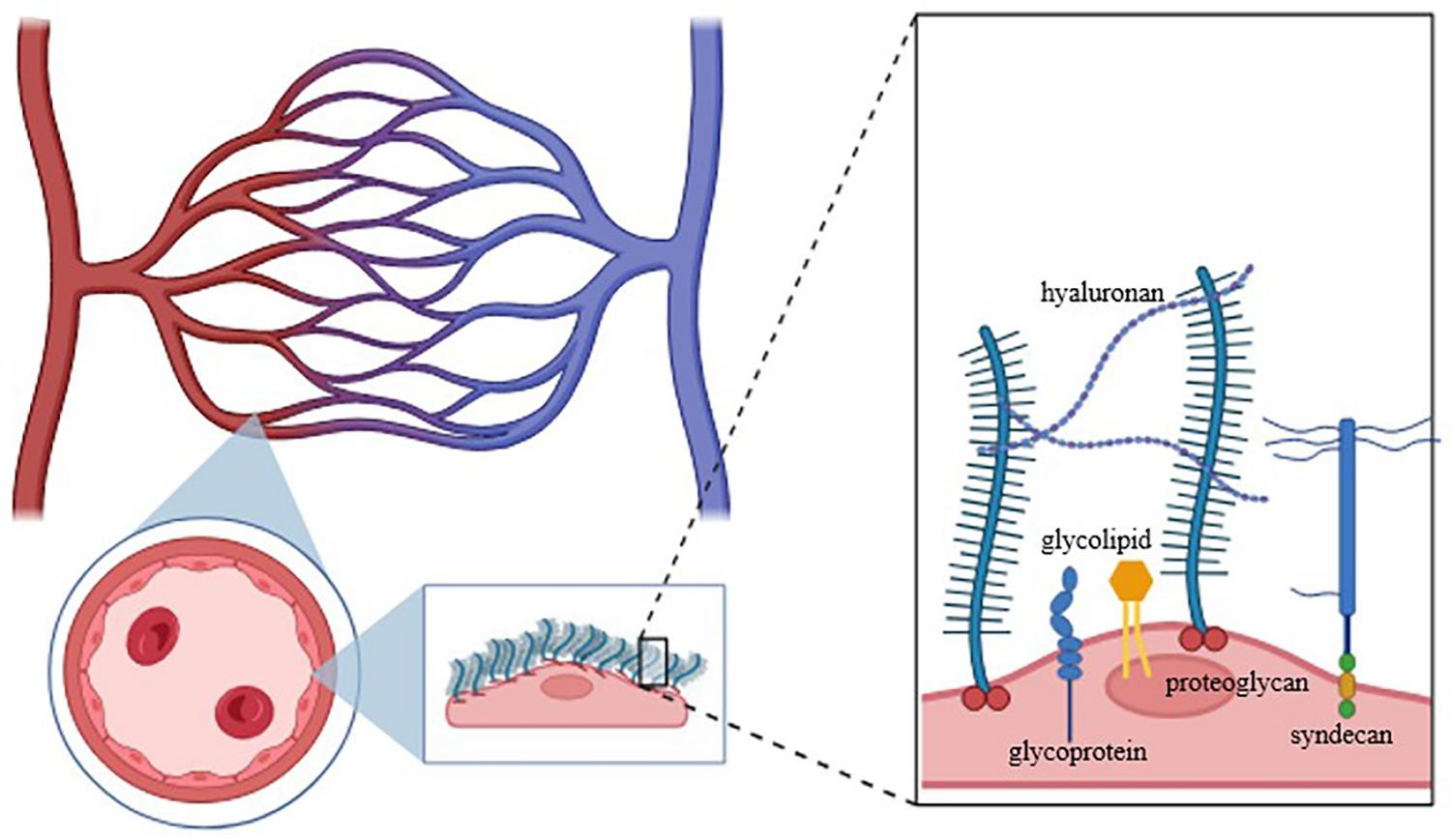
Capillary bed and microcirculation system. The illustration is zoomed in on a cross section of capillary vessel and depicts glycocalyx on endothelium. Created in https://BioRender.com.

In the heart and most other organs, oxygenated blood flows into arteries and progresses down to arterioles, capillaries, and lastly post-capillary venules before returning to the veins. The microcirculation, composed of arterioles, capillaries, endothelial glycocalyx, and venules, is the essential step in oxygenation of tissues (Figure 1). It is the direct source of oxygen delivery and extraction at the capillary level and also regulates solute exchanges between intravascular and tissue spaces, transports hormones and nutrients to tissues and mediates hemostasis and the immune system (7).

The capillaries are composed of a single layer of vascular endothelial cells that allow passage of individual red blood cells (RBCs). Endothelial cells are lined by the dynamic glycocalyx (Figure 1), a gel-like layer composed of glycosaminoglycans, proteoglycans, and plasma proteins such as antithrombin and superoxide dismutase (7). The glycocalyx plays a critical role in controlling permeability, oncotic gradients, and leukocyte-endothelial interactions (8). Inflammation and ischemia-reperfusion during cardiovascular surgery has been found to cause damage to both RBCs and the glycocalyx, leading to impaired vasoregulation and ultimately microcirculatory dysfunction. Proper functioning of the microcirculation is ultimately vital to the overall organ function on a macro scale.

## 3 Pathophysiology: how CPB disrupts microcirculation

### 3.1 Endothelial dysfunction and glycocalyx shedding

Endothelial injury during CPB arises from a combination of ischemia–reperfusion, abnormal shear stress, inflammation activation, and direct exposure of blood to artificial circuit surfaces. Among these insults, ischemia-reperfusion is the earliest and most dramatic driver of glycocalyx degradation. The glycocalyx is composed of membrane-bound proteoglycans and glycosaminoglycans that bind plasma proteins and retain plasma within the vascular lumen, and is the primary determinant of vascular barrier competence (9–12). In a landmark clinical study, Rehm et al. demonstrated that reperfusion, even after short periods of ischemia, produced massive shedding of the endothelial glycocalyx, with syndecan-1 and heparan sulfate increasing up to 42-fold and 10-fold after global ischemia, and even higher after regional ischemia associated with CPB (13). Electron microscopy in the same study confirmed structural destruction of the endothelial surface layer within minutes of reperfusion, establishing the glycocalyx as a highly vulnerable interface during cardiac surgery (13). These rapid biochemical and structural changes trigger microvascular instability that persists throughout the perioperative period.

Subsequent studies have demonstrated the functional consequences of this shedding. Wu et al. studied 30 cardiac surgery patients and observed that syndecan-1, heparan sulfate, and hyaluronan rose sharply after aortic unclamping, peaking approximately 1 h after CPB; these changes coincided with significant declines in perfused vessel density, demonstrating a significant association between glycocalyx degradation and microvascular hypoperfusion (14). Similarly, Robich et al. found that longer CPB duration correlated strongly with higher syndecan-1 levels and increased postoperative neutrophil mobilization, suggesting that glycocalyx fragments act not only as markers of injury but also as biologically active mediators that recruit inflammatory cells (15). Furthermore, longitudinal clinical data indicate that glycocalyx injury and associated microcirculatory disturbances can persist beyond the intraoperative period in patients undergoing CPB. In adults with on-pump CABG, a study found that initiation of CPB is followed by an immediate decrease in sublingual microvascular perfused vessel density and proportion of perfused vessels, and these disturbances have been shown to remain impaired throughout the first three postoperative days (16). During this interval, circulating markers of endothelial glycocalyx shedding, including syndecan-1 and heparan sulfate, are elevated, along with thinning of glycocalyx compared to pre-bypass values. These findings suggest that CPB-associated endothelial glycocalyx injury is not rapidly reversible and may contribute to delayed recovery of microvascular perfusion even after the restoration of systemic hemodynamics. Together, these findings identify glycocalyx shedding as a dynamic, mechanistically relevant event that shapes microcirculatory behavior during and after bypass.

In addition to ischemia-reperfusion phenomena, CPB exposes the endothelium to non-physiological shear forces and biomaterial contact, both of which contribute to further injury. Asberg and Videm demonstrated that contact with roller pumps and synthetic tubing could activate neutrophils by upregulating Mac-1 (CD11b/CD18), shedding L-selectin, and inducing aberrant adhesion patterns (17). Activated neutrophils subsequently release proteases and reactive oxygen species that degrade glycocalyx components and damage the endothelium (17). These shear-dependent and biomaterial-dependent perturbations act in concert with ischemia-reperfusion to amplify endothelial dysfunction.

Pre-existing endothelial vulnerability is an additional consideration. Patients undergoing cardiac surgery often have other comorbidities, such as hypertension, hyperlipidemia, and diabetes, that manifest in chronic inflammation and reduced bioavailability or sensitivity to nitric oxide (NO). Knežević et al. emphasized that this baseline dysfunction can lead to partial degradation of the glycocalyx preoperatively, making the endothelium more susceptible to CPB-related mechanical and biochemical injury (18). This preoperative fragility likely explains why some patients develop more profound postoperative microcirculatory dysfunction than others.

Experimental studies offer further insight into the hemodynamic consequences of endothelial damage. In a rat model of CPB, Dong et al. observed pronounced arteriolar vasoconstriction, reduced functional capillary density, perfusion heterogeneity, and severe albumin leakage into the interstitium, none of which recovered within 120 min after CPB termination (19). Loss of glycocalyx integrity impairs shear-stress mechanotransduction, diminishes NO-mediated vasodilation, increases vascular permeability, and promotes tissue edema—each of which contributes to impaired oxygen delivery and microvascular maldistribution. Collectively, the evidences from clinical and experimental literature describe a multi-hit process of endothelial injury in which glycocalyx shedding serves as both a marker of injury and a mechanistic amplifier of microvascular dysfunction during CPB.

### 3.2 Inflammatory activation and oxidative stress

CPB induces systemic inflammatory response through activation of multiple overlapping pathways, including contact activation and recruitment of leukocytes, complement cascade activation, and endotoxemia. The inflammatory response begins at the moment blood contacts the non-endothelialized surfaces of the extracorporeal circuit. Paparella et al. described how factor XII activation triggers the kallikrein–kinin cascade, complement activation (C3a and C5a), and intrinsic coagulation, resulting in early endothelial activation and leukocyte chemotaxis in response to NF-kB (20). Complement-derived anaphylatoxins further amplify inflammation by promoting neutrophil adhesion, mast-cell degranulation, and increasing vascular permeability. Banerjee et al. similarly highlighted that hypothermia, hemodilution, altered flow patterns, and supraphysiologic oxygen tensions during CPB exacerbate endothelial stress and stimulate cytokine release (21). These perturbations, together with the mechanical trauma of extracorporeal circulation, collectively prime the inflammatory response.

Among the multitude of initial inflammatory responses related to CPB, neutrophils play a key role. Asberg and Videm demonstrated that contact with biomaterial surfaces alone is sufficient to upregulate neutrophil integrins, diminish rolling behavior, and promote firm adhesion, thereby predisposing neutrophils to exaggerated inflammatory responses once they encounter the endothelium *in vivo* (17). Activated neutrophils release potent proteolytic enzymes such as elastase and myeloperoxidase, as well as reactive oxygen species (ROS) which degrade the endothelial glycocalyx and disrupt intercellular junctions (17). These events further increase vascular permeability and propagate microvascular injury.

In addition to the initial inflammatory responses triggered by nonendothelialized surfaces of CPB, ROS further compound microcirculation dysfunction with the addition of oxidative stress. Zakkar et al. described how ischemia leads to ATP depletion and hypoxanthine accumulation, while reperfusion enables the xanthine oxidase system to generate large quantities of ROS, including superoxide, hydrogen peroxide, hydroxyl radicals, and peroxynitrite (22). These species damage endothelial DNA, lipids, and proteins, reduce NO availability, and stimulate NF-*κ*B activation, which drives additional pro-inflammatory cytokine expression. The combined effects of ROS and cytokines produce a state of vasomotor dysregulation characterized by increased vasoconstriction, heterogeneous perfusion, and impaired microvascular oxygen delivery.

Cytokine surges represent another hallmark of CPB-induced inflammation. Paparella et al. documented rises in TNF-α, IL-6, IL-8, and IL-10 after CPB, contributing to endothelial injury, aberrant vasoregulation, and multiorgan dysfunction (20). TNF-α disrupts tight junctions and promotes endothelial apoptosis, while IL-6 correlates with postoperative morbidity and drives acute-phase responses. IL-8 is a potent neutrophil chemoattractant that propagates inflammatory cell recruitment into vulnerable microvascular beds. Endotoxemia, arising from splanchnic hypoperfusion and gut bacterial translocation, further amplifies inflammation by stimulating toll-like receptor–mediated cytokine release (20).

Finally, glycocalyx degradation itself contributes to inflammatory amplification. Robich et al. observed that circulating syndecan-1 can mobilize neutrophils from bone marrow, demonstrating that glycocalyx fragments serve as biologically active molecules that enhance systemic inflammation during CPB (15). Thus, endothelial injury and inflammation form a positive feedback loop, in which neutrophil activation, ROS production, cytokine release, and glycocalyx shedding collectively impair vasoregulatory function, disrupt microcirculatory flow, and contribute to postoperative organ dysfunction.

### 3.3 Hemodilution and functional shunting

Another contributing factor to microcirculatory dysfunction is hemodilution. Cardiopulmonary bypass requires priming of the extracorporeal circuit, which inevitably produces hemodilution, the dilution of blood components upon initiation of CPB. Mild hemodilution can be beneficial due to reduction in blood viscosity, increased flow in microcirculation, and decreased risk of thrombosis (23). However, moderate to severe hemodilution (e.g., hematocrit < 20%–25%) can impair oxygen-carrying capacity and contribute to functional shunting, a phenomenon where blood bypasses oxygen-extracting capillaries, resulting in tissue hypoxia (24). Therefore, there must be a balance between the reduced viscosity benefits and the impaired oxygen-carrying capacity to maximize patient care during surgery to maintain adequate systemic oxygen delivery and consumption during CPB (25).

The use of prime solutions in CPB is a major contributor to hemodilution, directly influencing oxygen delivery, microvascular perfusion, and postoperative outcomes. The standard prime volume (800–1,500 mL) significantly dilutes circulating blood upon bypass initiation, reducing hematocrit and oxygen-carrying capacity. Prime solutions are largely divided into two groups: crystalloid and colloid.

Crystalloid prime solutions, such as Ringer's lactate, Ringer's acetate, or normal saline, are the simplest and most widely used due to their low cost and ease of preparation. However, their low oncotic pressure promotes rapid transcapillary fluid shift and marked hemodilution, often reducing hematocrit by up to 40% and impairing oxygen delivery despite adequate pump flow (26). The resultant decrease in plasma colloid osmotic pressure contributes to interstitial edema and reduced capillary oxygen diffusion.

Colloid prime solutions include albumin and synthetic colloids like gelatin and hydroxyethyl starch. Compared to crystalloids, colloid prime solutions are found to preserve oncotic pressure and reduce fluid shifts (27). Additionally, albumin use has been associated with increased endothelial glycocalyx integrity, although the specific mechanism is still poorly understood (28). However, the high cost and potential severe perioperative anaphylaxis have limited the wide use of colloids (29). Despite the many theorized benefits of colloids mentioned above, large contemporary RCTs such as the PRIME trial and the ALBICS trial have yet to find differences in clinical outcomes of colloid prime solutions in CPB, when compared to crystalloid counterparts (30–32).

One strategy to mitigate the hemodilution is the use of retrograde autologous priming (RAP). RAP replaces part of the circuit's priming volume with the patient's own blood before initiating bypass, and can restore hematocrit levels by 3%–5% and improve oxygen delivery without increasing circuit resistance (33–35). However, whether this restoration of hematocrit from RAP will contribute to improved microcirculatory perfusion or improved clinical outcomes is still under investigation by a recent randomized trial, along with comparisons of other modern priming strategies such as recombinant colloids and combinations of colloid and crystalloids (36).

**Figure 2:**
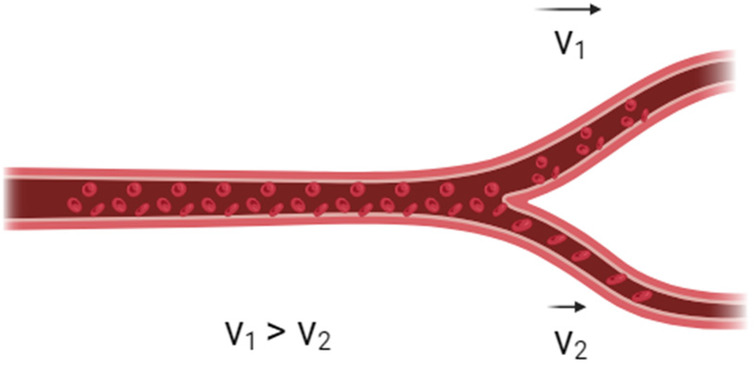
Network fåhræus effect. As shown in the illustration, RBCs preferentially enter microvascular branches with higher flow, leading to uneven distribution or shunting.

Excessive CPB-induced hemodilution not only reduces the oxygen content in the arterial system, but also impacts adequate oxygen delivery on capillary level due to maldistribution of flow from functional shunting (37). A key contributing mechanism is the network Fåhræus effect (Figure 2), which depicts the uneven distribution of RBCs through different microvasculature branches. When facing a bifurcation, RBCs preferentially enter higher-flow branches, while low-flow branches receive disproportionately fewer RBCs and more plasma. This leads to heterogeneous capillary hematocrit, where microvasculature with larger sizes carry RBC-rich blood while smaller capillaries are relatively RBC-depleted (38). In other words, when blood is hemodiluted, capillaries may face even lower hematocrit than the systemic flow, leaving surrounding tissues underperfused or intermittently perfused.

This arises as an issue in clinical practice, as oftentimes maldistribution of flow goes unnoticed throughout CPB, where hemodynamics appear adequate on the systemic level but patients still exhibit impaired oxygen extraction and regional ischemia. In den Os et al.'s study regarding CPB parameters associated with alterations in sublingual microcirculatory perfusion, they found that 77% of studies examining functional capillary density or perfused microvessel density have observed a significant decrease in microcirulatory perfusion (39). However, no effect of total vessel density or small vessel density was observed, demonstrating that while this microvascular network may remain intact, the maldistribution of flow causes dynamic dysfunction. Similar detrimental effects of maldistribution of flow in microvasculature have also been reported in cerebral and renal perfusions in patients undergoing CPB (40, 41). Clearly, this dissociation between systemic circulatory and microcirculatory perfusion remains a challenge in CPB management.

### 3.4 Microthrombosis and coagulopathy

Cardiopulmonary bypass, while essential for cardiac surgical procedures, activates coagulation cascade and induces microthrombosis, a result of the interplay between platelet activation, intrinsic and extrinsic coagulation pathways, and fibrin dynamics. These processes are initiated by exposure of blood to non-endothelialized CPB circuit surfaces, mechanical shear stress, and surgical trauma, thereby disrupting hemostatic regulation (20).

Platelet activation and dysfunction are among the earliest hemostatic changes during CPB. Contact with non-endothelialized circuit surfaces and shear stress from pumps induce platelet degranulation and activation, surface P-selectin expression, and platelet aggregation (42). Rinder et al. showed that CPB increases the level of activated platelets and promotes platelet–leukocyte conjugate formation via P-selectin, linking platelet activation by CPB to both thrombosis and inflammation (43).

In parallel, CPB triggers robust activation of the complement system via the classical, alternative, and lectin pathways. This results in the generation of anaphylatoxins (C3a, C5a) and membrane attack complex (C5b-9), which can cause direct damage to the endothelium, upregulate tissue factor expression, and enhance platelet activation and leukocyte adhesion (44). Consequently, complement-mediated endothelial injury and neutrophil activation are responsible for both microvascular plugging and consumption of coagulation factors that lead to microthrombosis and coagulopathy (45).

In a similar fashion, the activation of the intrinsic pathway is largely driven by contact of blood with the artificial surfaces of the CPB circuit. This contact activates factor XII and downstream kallikrein-kinin and coagulation pathways. The extrinsic pathway, in contrast, is primarily activated by tissue factor released from disrupted endothelium from operative trauma. This pathway may be especially relevant during periods of surgical manipulation and reperfusion, when thrombin generation further accelerates (46). Because heparin acts indirectly through antithrombin and is less effective against clot-associated thrombin and ongoing tissue factor–driven amplification, thrombin generation may continue via the extrinsic pathway despite apparently adequate anticoagulation. This explains that even though patients are systemically anticoagulated with heparin while on CPB, studies measuring prothrombin fragments and thrombin–antithrombin complexes have shown that thrombin generation persists throughout CPB. For instance, in patients undergoing coronary surgery on CPB, Knudsen et al. found that prothrombin fragment 1 + 2 and fibrinopeptide A continued to rise throughout bypass despite sustained heparin levels and antithrombin activity (47). Similarly, Ernofsson et al. demonstrated extensive thrombin generation during CPB in the setting of systemic anticoagulation with heparin (48).

The inadvertent activation of platelets and coagulation cascades produces microthrombosis in the microvasculature, contributing to regional organ injury. Platelet aggregates, fibrin deposits, and microthromboemboli occlude arterioles and capillaries, creating local malperfusion despite adequate systemic pump flow. Additionally, microthrombosis also provides a thrombogenic surface for platelet adhesion and fibrin deposition, further contributing to microthrombosis propagation. The effects of microemboli have been thoroughly studied in the setting of cerebral perfusion, where multiple studies have found that CPB circuit introduces microthromboemboli to the cerebral circulation, leading to micro-infarcts in the brain that could manifest in postoperative neurocognitive decline (49, 50). While the effect of microemboli on other organs following CPB are less systematically researched in humans, evidence from translational swine models nonetheless supports significant microemboli showering into other organs post-CPB, including lungs, liver, and skeletal muscle (51).

## 4 Measurement and monitoring of the microcirculation

Monitoring microcirculatory function during and after cardiac surgery with CPB presents a major challenge, but it remains critical for understanding how CPB-induced perturbations translate into tissue-level ischemia and subsequent organ dysfunction (1). Conventional systemic hemodynamic variables such as blood pressure, cardiac output, or global oxygen delivery often remain within acceptable ranges, even when perfusion at the microvascular level is severely compromised (52). In response, researchers have developed and refined complementary measurement techniques that target the microcirculation, including structural–functional imaging of microvessels, systemic biochemical markers of endothelial and glycocalyx integrity, and functional measures of tissue perfusion and oxygenation.

### 4.1 Imaging modalities

Handheld vital-microscopy (HVM) techniques remain the most direct and physiologically interpretable methods to assess microvascular perfusion in humans. These techniques permit real-time *in vivo* visualization of RBC flow through capillaries, arterioles, and venules, allowing quantification of microvascular density and flow dynamics (53). The first widely used method was orthogonal polarization spectral (OPS) imaging, which permitted sublingual microvascular observation without dyes or contrasts (54). Early clinical applications using OPS in cardiac surgery suggested that even within minutes of CPB initiation, sublingual microcirculation deteriorates, despite maintenance of global circulatory parameters (39). These early findings lay foundation for contemporary research by proving that CPB interrupts perfusion at the capillary level in a manner undetectable by standard intraoperative monitoring. However, OPS has important limitations, such as poor image resolution with motion or body fluid secretions and the need of an external light source (55). Consequently, while OPS was one of the first techniques developed in the field, it lacked the resolution and utility needed for robust modern clinical research.

Later, side-stream dark field (SDF) imaging emerged as a substantial advancement. SDF utilizes hemoglobin's absorption of green light to visualize flowing RBCs in microvessels (56). This approach delivers clearer and relatively more stable images than OPS under clinical conditions and allows quantitative analysis of microvascular parameters (55). From SDF video sequences, investigators derive metrics such as total vessel density (TVD), perfused vessel density (PVD), functional capillary density (FCD), small vessel density (SVD), proportion of perfused vessels (PPV), and microvascular flow index (MFI), which quantitatively describes flow quality across microvessels (57). The application of SDF in cardiac surgery has enabled the systematic study of CPB's impact on microvascular perfusion. For instance, a recent systematic review of 19 cardiac surgery studies using sublingual microscopy found that CPB led to significant reductions in FCD, PVD, or PPV in two thirds of the included studies (39). In many cases, these perfusion impairments persisted throughout surgery, and in some studies for at least 24 h postoperatively. Importantly, the same review noted that TVD and SVD often remained stable, and changes in MFI were inconsistent. This pattern suggests that CPB primarily reduces the fraction of microvessels that are perfused, rather than eliminating vessels altogether, meaning that structural microvascular density stays intact while flow becomes uneven or halted in a subset of vessels, further emphasizing the importance of modalities that can directly measure microvasculature flow.

Despite these strengths, SDF imaging has practical limitations that make widespread use challenging. First, reliable assessment typically requires multiple high-quality video sequences at each measurement time point, because microvascular flow can vary spontaneously and motion artifact or secretions can degrade image quality, and excluding poor-quality recordings may reduce sample size and introduce selection bias (58). Second, the limited resolution of SDF techniques hinders the detection of the small capillaries, particularly when flow is sluggish or intermittent (59). This limitation may lead to underestimation of baseline microvascular density and may exaggerate perceived reductions in perfusion when small capillaries fall below the detection threshold. Finally, SDF imaging captures only superficial microvascular beds such as the sublingual mucosa, which may not reflect perfusion in deeper organs such as the myocardium, kidney, or lung where CPB-induced microvascular dysfunction could be clinically critical (60).

To address some of these limitations, a newer imaging approach called incident dark field (IDF) imaging has been developed. IDF imaging integrates modern high-sensitivity sensors and improved optical components to enhance image contrast and resolution relative to SDF (59). In healthy human subjects, IDF has demonstrated detection of a greater number of capillaries than SDF, indicating a more accurate delineation of baseline microvascular architecture. Enhanced sensitivity is important because more complete visualization of the capillary network reduces the risk of overestimating impairment during CPB and increases the ability to detect subtle changes in perfusion or capillary recruitment. Additionally, IDF-derived leukocyte tracking has been applied in CABG patients, demonstrating that leukocyte–endothelium interactions can be visualized and quantified in the sublingual microcirculation during the peri-CPB period, allowing bedside microvascular monitoring to explore inflammatory responses associated with CPB (61).

Recent advances in HVM platforms and the integration of computer vision have further expanded microcirculatory assessment. Analysis now extends beyond vessel density and flow scoring to include quantitative measures of oxygen transport and inflammation. The OxyCam is a novel example that combines dark-field microscopy with multispectral imaging to estimate tissue red blood cell perfusion, tissue hematocrit, microvascular hemoglobin oxygen saturation, and tissue oxygen extraction (62). These parameters are particularly relevant in CPB, where macrocirculatory flow may appear preserved despite impaired microcirculatory oxygen delivery. Advanced computer-vision algorithms, such as MicroTools, further enhance analysis of microcirculatory data (63). They improve automated capillary detection and enable quantification of red blood cell velocity in high-resolution IDF sequences, thereby increasing sensitivity to subtle flow heterogeneity during CPB. While imaging provides real-time, structural, and functional insight into microcirculation, complementary systemic biochemical markers should also be employed to assess endothelial and glycocalyx integrity, inflammatory responses, and tissue perfusion at a molecular level.

### 4.2 Systemic biochemical markers

Given the technical challenges and anatomic restriction of imaging modalities, systemic biochemical markers have emerged as complementary tools for assessing microvascular and endothelial injury during CPB. Among these, circulating markers of endothelial glycocalyx degradation are the most extensively studied. Experimental and clinical data indicate that inflammatory mediators, oxidative stress, and mechanical forces during CPB promote shedding of glycocalyx components, thereby exposing the endothelial surface and impairing microvascular perfusion (64).

Prospective studies combining biochemical assays with microcirculatory imaging provide strong correlation between glycocalyx degradation and impaired microvascular perfusion during CPB. In adult cardiac surgery patients undergoing CPB, plasma concentrations of syndecan-1, heparan sulfate, and hyaluronan increased significantly during bypass and early reperfusion, particularly around the time of aortic declamping (18). Concurrent SDF imaging also demonstrated glycocalyx thinning and reduced perfused vessel density during the same period (65). Importantly, higher plasma concentrations of syndecan-1 and heparan sulfate correlated inversely with glycocalyx thickness and positively with microvascular permeability, suggesting a mechanistic association between glycocalyx loss and microcirculatory dysfunction rather than coincidental inflammatory response (66).

Beyond classic glycocalyx components, inflammatory cytokines such as interleukin-1β and proteolytic enzymes are additional biochemical markers that have been implicated as mediators of CPB-related endothelial injury (67). Of the proteolytic enzymes, matrix metalloproteinases are of particular interest, as they directly cleave glycocalyx components and may amplify endothelial injury through sustained proteolytic activity during and after CPB.^67^In addition to inflammatory cytokines and proteolytic enzymes, oxidative stress markers have been studied as indicators of CPB-induced microvascular injury. Increased production of ROS and accumulation of lipid peroxidation products during bypass contribute to endothelial dysfunction and glycocalyx degradation, as evidenced by early increases in circulating 8-isoprostane and nitrites/nitrates during on-pump cardiac surgery with CPB (68). Elevated oxidative stress has been associated with impaired microvascular perfusion, increased capillary permeability, and synergistic interaction with inflammatory cytokines to amplify endothelial injury (69). Therefore, measurement of oxidative stress biomarkers provides additional mechanistic insight into CPB-related microvascular compromise and complements assessments of glycocalyx and cytokine alterations.

Lastly, traditional systemic markers of hypoperfusion, including lactate, remain frequently used as indirect indicators of microcirculatory dysfunction after CPB. Hyperlactatemia may reflect impaired tissue oxygenation and correlate with increased postoperative morbidity and prolonged ICU stay (70). However, lactate levels are influenced by multiple non-microvascular factors, including hemodilution, hypothermia, catecholamine administration, and hepatic clearance (70, 71). Consequently, lactate should be interpreted as a functional surrogate of tissue perfusion rather than a specific marker of microvascular flow failure, and can be considered alongside biochemical markers of inflammation and oxidative stress for a more complete assessment of endothelial injury and microcirculatory compromise.

In addition to abovementioned systemic markers, organ-specific biomarkers have also been utilized to assess the level of microcirculatory dysfunction associated with CPB. In a randomized study comparing various CPB strategies by Aarts et al., cardiac (HFABP, troponin T, pro-BNP, and CPK), pulmonary (CC16, pneumoprotein), intestinal (IFABP), and hepatic (α-GST) biomarkers were collected and analyzed to demonstrate differential impacts of various CPB strategies on organ-specific microcirculation (72).

### 4.3 Functional tissue perfusion measures

While microvascular imaging and systemic biomarkers provide insight into microvasculature perfusion and integrity, they do not directly measure the functional consequences of microcirculatory disturbances, specifically tissue oxygen delivery and utilization. Functional perfusion monitoring therefore seeks to assess regional tissue oxygenation in real time. Among available techniques, near-infrared spectroscopy (NIRS) has gained widespread use in cardiac surgery due to its non-invasive nature and ability to provide continuous monitoring during CPB (73). NIRS estimates regional tissue hemoglobin oxygen saturation by exploiting the differential absorption spectra of oxygenated and deoxygenated hemoglobin in the near-infrared range (74). Since the signal originates predominantly from small vessels, including arterioles, capillaries, and venules, NIRS is often considered a surrogate marker of microvascular oxygenation (75, 76). Importantly, NIRS does not rely on arterial pulsatility and therefore remains usable during non-pulsatile CPB conditions (73). Clinically, NIRS is most commonly applied to monitor cerebral, renal, or peripheral tissue oxygenation during surgery and has been used to detect gross regional perfusion deficits such as lower limb ischemia during cardiac surgery (77, 78). To improve sensitivity to microvascular dysfunction, NIRS is frequently combined with dynamic testing, such as the vascular occlusion test (VOT). This maneuver involves transient arterial occlusion followed by reperfusion, allowing calculation of desaturation and resaturation slopes that may reflect oxygen utilization and microvascular reactivity (79).

However, clinical evidence supporting the ability of NIRS with VOT to detect CPB-induced microvascular dysfunction remains inconsistent. In a prospective study, peripheral NIRS with VOT failed to demonstrate reliable discriminative changes in microvascular function after cardiac surgery with CPB, with paradoxical postoperative tissue saturation increases, likely driven by hemodilution, decreased viscosity, and reduced metabolic demand (73). Several intrinsic limitations complicate interpretation of NIRS data. First, NIRS measures a composite signal from arterial, capillary, and venous compartments (75). As a result, intermittent microvascular flow impairment may be masked if well-perfused regions dominate the signal. Second, the accuracy of measurements is influenced by local tissue characteristics such as adiposity, probe placement, and skin pigmentation (80). Third, changes in metabolic demand strongly influence tissue oxygenation; during hypothermia or deep sedation, oxygen consumption decreases, potentially maintaining normal tissue saturation despite impaired microvascular flow (81).

Despite these limitations, NIRS retains clinical utility in selected scenarios. Continuous monitoring enables rapid detection of gross perfusion abnormalities, such as sustained cerebral desaturation during CPB or compromised peripheral perfusion after arterial cannulation (77). In pediatric cardiac surgery, renal NIRS values correlate with subsequent acute kidney injury, suggesting a potential role for continuous regional tissue oxygen monitoring in risk stratification (82). In adult cardiac surgery, combining NIRS with complementary modalities, such as intermittent microvascular imaging or serial biomarker measurements, may improve detection of clinically relevant microvascular dysfunction.

Beyond *in vivo* optical spectroscopy for regional oxygenation, studies of isolated microvessels from fresh tissue samples offer critical mechanistic insight into how CPB influences microvascular structure and function at the cellular level. Experimental animal models of CPB consistently demonstrate that microvascular perfusion impairment is not simply a global hemodynamic phenomenon but involves local endothelial injury, increased leukocyte adhesion, and functional capillary loss (19, 83, 84). For instance, in a rat model using fluorescent microscopy, intestinal microcirculation and leukocytes were visualized with fluorescent staining, and CPB was shown to significantly reduce functional capillary density, with concomitant vasoconstriction, reduced blood velocity, increased microvascular permeability, and sustained leukocyte adherence to the endothelium in splanchnic microcirculation (19).

Studies in a porcine model of cardioplegia and cardiopulmonary bypass have demonstrated changes in microvascular endothelial and smooth muscle dysfunction in the coronary (85, 86), pulmonary (87, 88), mesenteric (89), cerebral (90), and other vascular beds. These changes are in general corroborated from experiments from human tissues after surgery. For instance, *ex vivo* investigations of microvessels harvested from atrial appendage tissue during cardiac surgery have demonstrated that CPB alters microvascular reactivity and endothelial signaling pathways, including increased expression of stromal-derived factor-1α and resultant changes in microvessel contractile responses, which parallel *in vivo* microcirculatory perfusion disturbances seen clinically (91). Collectively, these mechanistic studies using fresh tissue and isolated microvessels validate the physiological relevance of *in vivo* observations from imaging and perfusion monitoring, illustrating how CPB, through inflammation, endothelial injury, and altered vasomotor control, contributes to functional microvascular impairment that cannot be monitored solely using global hemodynamic measures.

## 5 Cardioprotective strategies targeting microcirculation

Microcirculatory dysfunction during CPB arises from the combination of altered shear stress patterns, exposure of blood to artificial surfaces, ischemia-reperfusion injury, and activation of inflammatory and coagulation cascades that directly target the vascular endothelium (45). Although contemporary CPB management maintains global hemodynamic variables such as mean arterial pressure, pump flow, and systemic oxygen delivery within optimal ranges, a substantial body of experimental and clinical evidence demonstrates that these parameters do not reliably reflect perfusion at the level of microcirculation (92). Reductions in functional capillary density, increased spatial and temporal flow heterogeneity, impaired endothelial-dependent vasomotor control, and degradation of the endothelial glycocalyx have all been documented during and after CPB, even when systemic circulatory targets are achieved (93). These observations have driven the development of cardioprotective strategies that explicitly aim to preserve microvascular perfusion and endothelial integrity, rather than relying solely on optimization of global hemodynamics.

### 5.1 Perfusion and mechanical strategies

One such emerging advancement is pulsatile cardiopulmonary bypass. The rationale for pulsatile CPB is grounded in the recognition that endothelial cells are highly sensitive to the magnitude and temporal characteristics of shear stress (94). Under physiologic conditions, pulsatile flow generates oscillatory shear forces that regulate endothelial nitric oxide synthase (eNOS) activity, cytoskeletal organization, and transcriptional programs governing inflammation, coagulation, and barrier function (95). *In vivo* studies have demonstrated that exposure of endothelial monolayers to pulsatile shear stress increases nitric oxide production and suppresses expression of adhesion molecules, whereas steady or low-shear, non-pulsatile flow patterns induce increased vasoconstriction and heightened inflammatory response (96, 97). These mechanistic findings provided early justification for the potential benefits of pulsatile CPB.

Animal models offered the first opportunity to directly test this hypothesis at the microvascular level. In a neonatal piglet model using radioactive microspheres to quantify regional organ blood flow, pulsatile perfusion resulted in significantly higher cerebral blood flow across multiple brain regions and improved preservation of myocardial and renal perfusion compared with non-pulsatile perfusion, similar mean arterial pressures maintained during CPB between cohorts (98). In another canine CPB study, compared to non-pulsatile flow at equivalent mean flow rates, pulsatile flow resulted in significantly greater renal blood flow and improved renal metabolic parameters, indicating enhanced microvascular and end-organ perfusion with pulsatile perfusion (99). Together, these clinical findings show that the pattern of blood flow itself, in addition to the perfusion pressure, is also important for maintaining microvascular and organ perfusion during CPB.

Similar benefits of pulsatile CPB are also shown in clinical studies. In a systematic review of randomized trials including patients undergoing CPB surgery, Abdelraouf et al. reported higher creatinine clearance, a decrease in lactate levels, and a decrease in hospital stay in patients supported with pulsatile CPB compared with non-pulsatile flow (100). However, the authors also noted inconsistent effects across different trials, specifically in terms of renal outcomes, and attributed the heterogeneity to differences in the definition of pulsatility, perfusion techniques, and postoperative care protocols (101).

Another strategy to mitigate the deleterious effects of conventional CPB, particularly hemodilution, inflammatory activation, and microcirculatory impairment, is Miniaturized extracorporeal circulation (MECC) (102). An early meta-analysis of randomized controlled trials demonstrated that MECC, by incorporating closed circuits, reduced priming volumes, and biocompatible coatings, significantly reduced RBC transfusions in patients undergoing (103). More recent trials and meta-analysis on MECC have congruent results, where patients undergoing CPB with MECC have mitigated hemodilution and microcirculatory dysfunction, and sometimes even improved perioperative outcomes clinically (104). Despite the promising evidence from clinical trials and advocacy from the Minimal invasive Extra-Corporeal Technologies international Society (MiECTiS) since 2016, the adoption of MECC remains limited due to increased technical complexity and the need for specialized training and institutional experience (105).

Ultrafiltration is another alternative mechanical strategy aimed at mitigating microcirculatory dysfunction by reducing hemodilution, interstitial edema, and circulating inflammatory mediators generated during CPB (106, 107). Ultrafiltration, a mechanism to remove excess water and low-molecular-weight plasma contents designed for renal dialysis, was first introduced to cardiac surgery for use in conjunction of CPB in late 1970s (108). Over the last few decades, ultrafiltration has become more widely accepted in CPB as a strategy to hemoconcentrate and remove pro-inflammatory cytokines during bypass.

Pediatric cardiac surgery provided early evidence supporting this approach. In a review on ultrafiltration in the context of pediatric cardiac surgery, Bierer et al. found that across several studies, ultrafiltration significantly increased myocardial and pulmonary function and reduced inflammatory cytokines, fluid overload, and bleeding complications (109). In adult cardiac surgery, a clinical trial showed that ultrafiltration is associated with reductions in postoperative weight gain, transfusion requirements, and morbidity (110). However, another single-center study showed that ultrafiltration may have limitations as well, noting that conventional ultrafiltration is a potential risk factor for acute kidney injury after surgery involving CPB (111). These findings suggest that while ultrafiltration may attenuate edema and reduce inflammatory burden, it may come with risks as well, especially in the context of renal function, highlighting the need for additional strategies to protect the endothelium and preserve capillary perfusion.

Leukocyte depletion filters have been investigated as a means of limiting neutrophil-mediated microvascular injury during CPB. Activated neutrophils contribute to endothelial damage through release of reactive oxygen species, proteases, and pro-inflammatory cytokines, and can physically obstruct capillaries, leading to heterogeneous flow and functional capillary loss (112). A prospective randomized study demonstrated that early leukocyte depletion reduces leukocyte-endothelial adhesion, decreases IL-10 serum levels, and preserves lung function temporarily after CPB (113). In another clinical trial, Gu et al. reported improved postoperative pulmonary gas exchange and reduced inflammatory markers in patients supported with leukocyte-depleted CPB circuits (114). However, rapid activation of neutrophils upon initiation of CPB may limit the efficacy of filtration strategies introduced after the activation of inflammatory cascades.

Circuit coatings and biocompatible surfaces are another strategy aiming to reduce blood-surface interactions that trigger complement activation, coagulation, and endothelial injury (115). In particular, heparin-coated circuits have been shown to attenuate complement activation and cytokine release (116). A clinical study by Dekker et al. demonstrated lower circulating concentrations of glycocalyx degradation markers, including syndecan-1 when heparin-coated circuits are used (117). In addition, the authors found that when heparin-coated circuits are used, there are smaller increases in perfused boundary region, consistent with partial preservation of glycocalyx thickness.

### 5.2 Ischemic and pharmacologic conditioning

Ischemic conditioning strategies exploit endogenous protective signaling pathways activated by brief, non-lethal episodes of ischemia (118). At the molecular level, ischemic preconditioning activate intracellular cascades such as the reperfusion injury salvage kinase (RISK) and survivor activating factor enhancement (SAFE) pathways, which act to mitigate oxidative stress, inhibit opening of the mitochondrial permeability transition pores, and preserve cellular viability (119). These pathways operate on endothelial cells as well as cardiomyocytes, where they modulate nitric oxide bioavailability, inflammatory signaling, and barrier function. Pre-clinical studies have demonstrated that ischemic conditioning of the heart preserves coronary microvascular reactivity, reduces no-reflow phenomenon, and maintains functional capillary density following ischemia-reperfusion injury (120–122).

Remote ischemic preconditioning, a strategy that temporarily restricts blood flow to a distant organ or tissue (e.g., limbs), has attracted particular interest in cardiac surgery because it can be applied non-invasively prior to the CPB initiation. Kharbanda et al. demonstrated that transient upper-limb ischemia resulted in increased endothelium-dependent vasodilation in healthy volunteers, providing early evidence of systemic endothelial protection mediated by circulating or neural signals (123). Subsequent translational animal studies showed that remote ischemic preconditioning preserved capillary perfusion and reduced leukocyte adhesion in myocardial and skeletal muscle microcirculation during CPB (124, 125). In early randomized clinical studies, remote ischemic preconditioning was associated with reductions in postoperative myocardial injury. Thielmann et al. reported lower postoperative cardiac troponin I concentration in CABG patients who received remote ischemic preconditioning prior to CPB compared to patients who did not receive remote ischemic preconditioning, despite both groups having equivalent preoperative troponin concentrations (126). However, larger multicenter trials did not reproduce these effects. The ERICCA and RIPHeart trials, which enrolled broad cardiac surgical populations, found no reduction in troponin release or major adverse cardiac events with remote ischemic preconditioning (127). Nevertheless, the absence of microcirculatory endpoints in these trials leaves a question about whether remote ischemic preconditioning exerted endothelial or capillary-level benefits that did not translate into observable clinical benefit.

### 5.3 Pharmacologic approaches

Pharmacologic strategies targeting myocardial injury and microvascular dysfunction have emerged as an important adjunct to traditional cardioprotective techniques in cardiac surgery. Given that CPB induces global ischemia–reperfusion injury characterized by oxidative stress, inflammation, calcium dysregulation, and endothelial dysfunction, pharmacologic interventions have largely focused on modulating these pathways to preserve microvascular integrity and myocardial performanc (128).

With a similar concept to ischemic preconditioning, emerging pharmacologic preconditioning strategies seek to activate similar protective pathways without inducing ischemia. Volatile anesthetics such as sevoflurane and isoflurane activate mitochondrial potassium channels and RISK signaling, demonstrating myocardial and endothelial protection in experimental models (129, 130). These agents may attenuate ischemia–reperfusion injury by reducing oxidative stress and modulating inflammatory responses; however, clinical data remain mixed, with some studies demonstrating reductions in biomarkers such as troponin release and others showing no significant improvement in clinical outcomes (128). Similarly, NO, a key endothelial-derived mediator, plays a central role in maintaining coronary microvascular tone and reducing leukocyte adhesion and platelet aggregation. Augmentation of NO signaling has been proposed as a therapeutic strategy to counteract CPB-induced endothelial dysfunction, although its clinical application remains variable (128).

Statins represent one of the most widely studied pharmacologic classes in perioperative cardioprotection due to their pleiotropic effects on inflammation, endothelial function, and oxidative stress. While statins have been shown to improve eNOS activity and reduce vascular inflammation, its perioperative efficacy for CPB-related microcirculatory dysfunction is still under debate. Preclinical translational models have shown that pre-CPB statin loading could attenuate CPB-induced myocardial inflammatory injury, promote NO-dependent coronary relaxation, and improve cardiac function (131, 132). Despite the mechanistic plausibility and preclinical success, majority of clinical trials have shown no difference in postoperative outcomes in patients who received preoperative statin treatment. In a systematic review that included 8 RCTs, Antunes et al. concluded that preoperative statin treatment was not associated with a reduction in adverse clinical outcomes after cardiac surgery (133).

As an anti-inflammatory agent, corticosteroids have had persuasive mechanistic rationale in microcirculatory protection after CPB, as they can attenuate inflammation, endothelial activation, and ischemia–reperfusion injury. Early mechanistic studies did show reduced cardiac troponin I release with methylprednisolone and decreased glycocalyx shedding with hydrocortisone, but further effects on clinical outcomes were inconsistent (134–136). Larger randomized trials have not demonstrated meaningful clinical benefit. The DECS trial showed no reduction in major adverse events despite shorter ICU stay, while the SIRS trial found no improvement in mortality or major morbidity and reported increased myocardial injury with steroids (137, 138). A recent systematic review and meta-analysis concluded that corticosteroids provide little or no mortality benefit and have uncertain effects on organ protection (139). Overall, current evidence does not support routine use, and steroids are now considered for selective rather than prophylactic application in adult cardiac surgery.

Oxidative stress is a central driver of myocardial and microvascular injury during CPB, and antioxidant therapies such as vitamin C and N-acetylcysteine have therefore been investigated as potential adjuncts to reduce oxidative burden. Clinical evidence suggests that antioxidant supplementation may reduce postoperative complications such as atrial fibrillation, likely through attenuation of oxidative stress and inflammation, although the magnitude of benefit varies across studies (140).

More recently, cardiometabolic agents such as sodium–glucose cotransporter-2 (SGLT2) inhibitors, glucagon-like peptide-1 (GLP-1) receptor agonists, and dipeptidyl peptidase-4 (DPP-4) inhibitors have gained attention for their potential cardioprotective and microvascular effects. SGLT2 inhibitors, in particular, have demonstrated favorable effects on myocardial energetics, inflammation, and endothelial signaling. In a swine model of chronic myocardial ischemia, canagliflozin treatment was associated with proteomic changes indicative of improved metabolic and vascular signaling pathways, suggesting a role in enhancing myocardial adaptation to ischemic stress (141). Additionally, clinical and experimental data indicate that SGLT2 inhibitors and GLP-1 receptor agonists may improve microvascular function and reduce cardiovascular events, potentially through effects on oxidative stress, endothelial function, and inflammatory signaling (142). DPP-4 inhibitors have also been shown to modulate microvascular complications, although their direct role in perioperative cardioprotection is less well established (142).

Pharmacologic cardioprotection represents a multifaceted approach targeting key mechanisms of myocardial and microvascular injury during CPB. While agents such as volatile anesthetics, statins, antioxidants, and cardiometabolic therapies demonstrate promise, their effects are often context-dependent and may not consistently translate into improved clinical outcomes. Future investigations integrating mechanistic insights with clinically meaningful endpoints will be essential to optimize pharmacologic strategies for perioperative cardioprotection.

## 6 Limitations, gaps, and future research

Important gaps remain in how CPB-related microcirculatory dysfunction is modeled, measured, and translated into effective perioperative therapies clinically. A major limitation is the translational gap between preclinical animal models and human CPB physiology. Although small-animal models enable detailed mechanistic study, they differ substantially from clinical cardiac surgery with CPB in blood volume utilized, hemodynamics, perfusion duration, and survival conditions. In addition, isolated ischemia-reperfusion models do not fully capture the heterogeneous responses seen in patients with comorbidity such as diabetes, pre-existing cardiovascular disease, chronic kidney disease, and aging. More representative models are needed to better reflect human inflammation, microcirculation in organs, and perioperative complexity.

Standardized measurement and quantification of microcirculatory dysfunction remain another major barrier. Techniques such as SDF imaging, OPS, and NIRS have provided valuable insight into microvascular perfusion, but their use is restricted by inter-device variability, lack of standardized acquisition and analysis, and limited access to deeper tissue and organs (143). As a result, microcirculatory endpoints are used inconsistently in clinical trials and rarely integrated into routine clinical practice, making comparison across studies on microcirculation difficult.

Patient heterogeneity is also insufficiently addressed. Older adults and patients with reduced ejection fraction, chronic kidney disease, or heightened inflammatory states appear particularly vulnerable to endothelial injury and microcirculation dysfunction yet remain underrepresented in both preclinical animal models and clinical trials (144). Future work should better define subgroup-specific risk profiles and therapeutic windows.

Collectively, these limitations highlight the need for standardized microcirculatory endpoints, translationally relevant models, mechanistic studies in high-risk subgroups, and the incorporation of advanced molecular technologies. Opportunities for integrating advanced imaging, metabolomics, and proteomic profiling show promise to bridge these gaps. High-throughput metabolic profiling has already demonstrated the ability to capture dynamic biochemical signatures of ischemia–reperfusion injury in patients undergoing cardiac surgery (145). Coupling high-throughput multi-omic data with microcirculatory characterization and quantification could allow researchers to correlate nuance yet discrete metabolic states with perfusion deficits, generating a systems-level understanding of endothelial injury and microcirculatory dysfunction in various organs. Future research should also include longitudinal human datasets and design interventional trials that explicitly incorporate microcirculation as endpoints. Only through such integrated, multimodal approaches can the field progress toward adequate microcirculatory monitoring and management during CPB and improve postoperative organ protection.

## 7 Conclusion

Microcirculatory dysfunction is a key yet under-recognized contributor of morbidity after cardiac surgery with CPB, occurring despite preserved systemic hemodynamics. Endothelial injury, glycocalyx shedding, inflammation, hemodilution, and microthrombosis lead to impaired microcirculation, resulting in tissue hypoxia that is not adequately captured by conventional monitoring. Although multiple cardioprotective strategies show biological promise in translational models, clinical evidence for improving microvascular perfusion and outcomes remains inconsistent. Future work should focus on standardizing quantifiable microcirculatory endpoints and improving translational models to accurately depict microcirculatory dysfunction after CPB, to ensure more successful clinical applications of cardioprotective strategies.
